# *In situ* generated D-peptidic nanofibrils as multifaceted apoptotic inducers to target cancer cells

**DOI:** 10.1038/cddis.2016.466

**Published:** 2017-02-16

**Authors:** Xuewen Du, Jie Zhou, Huainin Wang, Junfeng Shi, Yi Kuang, Wu Zeng, Zhimou Yang, Bing Xu

**Affiliations:** 1 Department of Chemistry, Brandeis University, Waltham, MA 02454, USA; 2 The Heller School for Social Policy and Management, Brandeis University, Waltham, MA 02454, USA; 3 The College of Life Sciences, Nankai University, Tianjin 300071, China

## Abstract

Nanofibrils of small molecules, as a new class of biofunctional entities, exhibit emergent properties for controlling cell fates, but the relevant mechanism remains to be elucidated and the *in vivo* effect has yet to be examined. Here, we show that D-peptide nanofibrils, generated by enzyme-instructed self-assembly (EISA), pleiotropically activate extrinsic death signaling for selectively killing cancer cells. Catalyzed by alkaline phosphatases and formed *in situ* on cancer cells, D-peptide nanofibrils present autocrine proapoptotic ligands to their cognate receptors in a juxtacrine manner, as well as directly cluster the death receptors. As multifaceted initiators, D-peptide nanofibrils induce apoptosis of cancer cells without harming normal cells in a co-culture, kill multidrug-resistant (MDR) cancer cells, boost the activities of anticancer drugs, and inhibit tumor growth in a murine model. Such a supramolecular cellular biochemical process (consisting of reaction, assembly, and binding) for multi-targeting or modulating protein–protein interaction networks ultimately may lead to new ways for combating cancer drug resistance.

Kinases and phosphatases regulate phosphorylation and dephosphorylation,^1, 2^ respectively, and control a variety of cellular processes. Kinase inhibitors have achieved relative successes in chemotherapy, but the development of phosphatases inhibitors remains to be a challenge. Since a number of phosphatases (e.g., PTEN^3^) are important tumor suppressors, the lack of selectivity of phosphatase inhibitors is a major concern. The inherent difficulty for developing specific phosphatase inhibitors has earned phosphatases the reputation of undruggable.^4^ Thus, new approaches to make phosphatases druggable are needed. Departing from the dogmatic approach of enzyme inhibition, we have been exploring the use of alkaline phosphatase (ALP) to catalyze the formation of molecular nanofibrils via self-assembly^5, 6^ for killing cancer cells.^7, 8, 9, 10, 11, 12^ The merit is that nanofibrils of small molecules, spatiotemporally defined by enzymatic catalysis and self-assembly (i.e., EISA), can interact with multiple cellular proteins and interrupt multiple cellular processes to kill cancer cells selectively,^13, 14^ even without inducing acquired drug resistance.^13^ After we demonstrated that EISA-generated nanofibrils selectively kill cancer cells,^15, 16^ several labs have already validated this concept of EISA in cell assays.^17, 18^ However, the relevant molecular and cellular mechanisms of cell death induced by the nanofibrils remain to be elucidated, and the *in vivo* effects of the nanofibrils generated by EISA have yet to be examined.

Here, we report the mechanistic study of using ALP catalysis, not ALP inhibition, to generate molecular nanofibrils for selectively killing cancer cells. Specifically, ALP, as an ectoenzyme,^19^ catalyzes the formation of pericellular nanofibrils of D-tripeptides (DTPs) on cancer cells, which pleiotropically activate cell death signaling by presenting autocrine death ligands (e.g., TNF-*α*^20^ and TRAIL^21^) to their cognate receptors (e.g., TNFR1/2, DR3/4/5)^22^ in a juxtacrine manner,^23^ as well as directly clustering the receptors (e.g., CD95^24^). As the localized multifaceted initiators, DTP nanofibrils induce apoptosis.^25^ The ALP-generated DTP nanofibrils kill cancer cells without harming normal cells in a mimicked tumor microenvironment,^26^ kill multidrug-resistant (MDR) cancer cells, boost the activities of anticancer drugs (e.g., cisplatin^27^ and NF-*ĸ*B inhibitors^28, 29^), and inhibit tumor growth in a murine model. These results demonstrate a paradigm shift from inhibition to reaction, that is, the use of a multiple step – reaction, assembly, and binding – process^30^ to modulate protein–protein interaction networks for selectively killing cancer cells.

## Results and Discussion

### ALP-generated nanofibrils for killing HeLa cells in a co-culture

**Figure 1:**
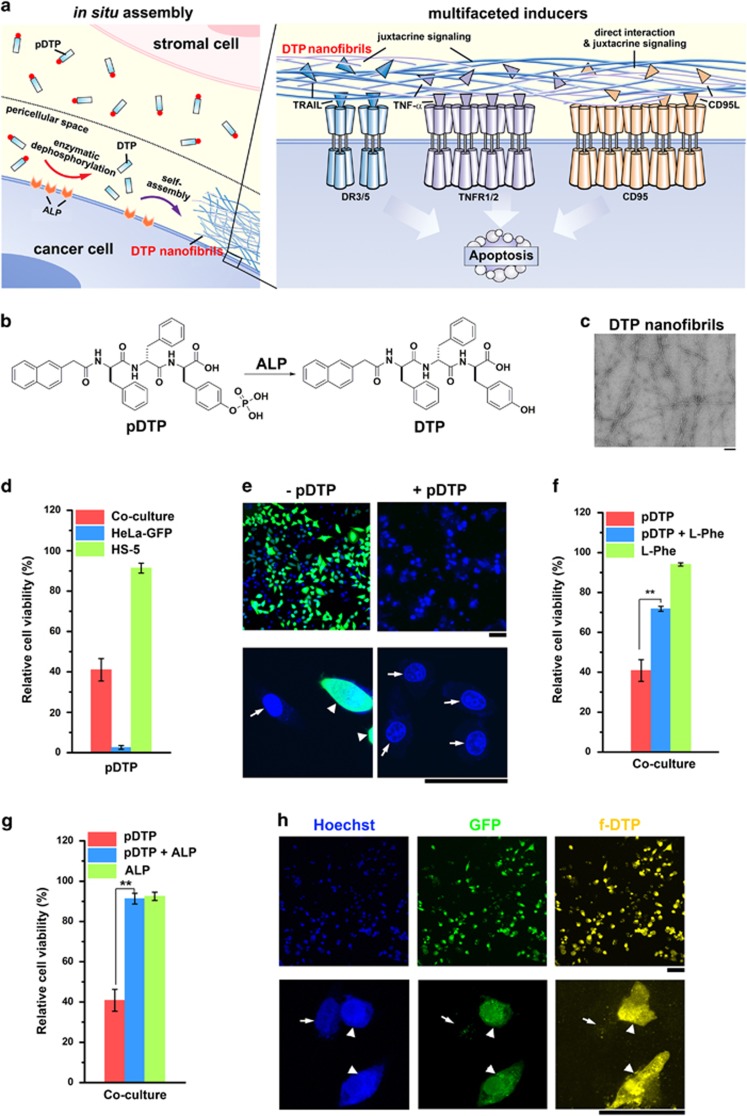
Enzyme *in situ* generates pericellular D-peptide nanofibrils for selectively inhibiting cancer cells in co-culture. (**a**) The illustration of the pericellular DTP nanofibrils formed by enzyme-instructed self-assembly (EISA) to selectively inhibit cancer cells in co-culture via promiscuously activating cell death signaling. (**b**) Chemical structures of the precursor (pDTP), the self-assembly tripeptide (DTP), and the dephosphorylation of the precursor catalyzed by PLAP. (**c**) Transmission electron microscopic (TEM) images of DTP nanofibrils formed by the addition of alkaline phosphatase (5 U/ml) to the solution of pDTP at the concentration of 362 *μ*g/ml. (**d**) Relative cell viability of the co-cultured cells (HS-5 and HeLa-GFP cells), HeLa-GFP, or HS-5 incubated with pDTP. The initial number of cells is 1.0 × 10^4^/well (e.g., 1.0 × 10^4^ HeLa-GFP or HS-5 cells, or mixture of 5.0 × 10^3^ HeLa-GFP cells and 5.0 × 10^3^ HS-5 cells) and [pDTP]=216 *μ*g/ml. (**e**) The fluorescent images of low (top) or high (bottom) magnification of co-cultured cells incubated with or without pDTP (362 *μ*g/ml) for 48 h. Blue indicates all the live cells stained by Hoechst 33342 (1 *μ*g/ml, 5 min) before fluorescent imaging; green indicates the live HeLa-GFP cells. The initial number of cells is 1.0 × 10^5^/well. Relative cell viability of the co-cultured HS-5 and HeLa-GFP cells (**f**) incubated with pDTP, pDTP+L-Phe, or L-Phe, or (**g**) incubated with pDTP, pDTP+AP, or AP in DMEM for 48 h. The initial number of cells is 1.0 × 10^4^/well and [pDTP]=216 *μ*g/ml, [L-Phe]=1.0 mM, [AP]=0.1 U/ml. *n*=3. Data are shown as mean±S.D. *P<0.05, **P<0.01 by Student's *t*-test. (**h**) The fluorescent images of low (top) or high (bottom) magnification of co-cultured cells incubated with f-pDTP (493 *μ*g/ml) for 4 h. Blue indicates all the live cells stained by Hoechst 33342; green indicates the live HeLa-GFP cells; and yellow indicates the peptide nanofibrils of f-DTP. The initial number of cells is 1.0 × 10^5^/well. The scale bars are 100 nm in (**c**), 100 *μ*m in (top of (**e**) and (**h**)), 50 *μ*m in (bottom of (**e**) and (**h**)). Bottom row of (**e**) and (**h**): white arrows point at HS-5, and arrow heads at HeLa-GFP

Since certain ALP (e.g., placental alkaline phosphatase (PLAP) on HeLa cells^31^) is overexpressed on the surface of certain cancer cells in the co-culture (mixture of cancer (HeLa) and normal (HS-5) cells), the precursor of DTP (i.e., a phosphorylated DTP, pDTP) is dephosphorylated locally on the surface of the cancer cells to generate DTP nanofibrils (Figure 1a). Acting as a pericellular nanonet just around the cancer cells, the DTP nanofibrils present the secreted, different proapoptotic ligands (e.g., TNF-*α*, CD95L, and TRAIL) from cancer cells to bind with different extrinsic cell death receptors (e.g., TNFR1/2, CD95, and DR3/4/5), or directly interact with the death receptors (e.g., CD95), which leads to the death of cancer cells only. Multiple evidences in the following sections support the mechanism illustrated in Figure 1a.

Adding ALP to the solution of pDTP (Figure 1b) results in DTP nanofibrils (Figure 1c) (diameters of 7±2 nm), indicating that ALP overexpressed on the cancer cells can generate DTP nanofibrils. Adding pDTP in the co-culture of HeLa and HS-5 (as a mimic of tumor microenvironment^26^) selectively kills the cancer cells (HeLa-GFP^32^) in the co-culture (Figure 1d and Supplementary Figure 1a), agreeing with the results in their separated cultures (Supplementary Table 1). As shown in the fluorescent imaging (Figure 1e and Supplementary Figure 1b) of the co-culture treated with pDTP, contrasting to the control, almost no HeLa-GFP cells live, while the HS-5 cells remain viable. The addition of L-phenylalanine (L-Phe), an uncompetitive inhibitor of PLAP,^33^ almost doubles the viability of HeLa-GFP cells, in comparison with only pDTP in culture medium (Figure 1f and Supplementary Figure 1c), excluding the possibility that pDTP acts as surfactants to lyse cells and confirming that PLAP on HeLa cells catalyzes the EISA of pDTP to selectively kill cancer cells. siRNA knock-down of PLAP on HeLa cells protects the cells from pDTP, confirming that PLAP is essential for killing HeLa cells (Supplementary Figure 1e) by EISA. Simultaneous addition of exogenous ALP with pDTP abrogates the cytotoxicity of pDTP (Figure 1g and Supplementary Figure 1d). Meanwhile, we design a fluorescent pDTP (f-pDTP) (Supplementary Figures 2a and b), which is innocuous to cells (likely due to f-DTP resulting in nanofibrils different from DTP nanofibrils) (Supplementary Figure 2c), for imaging the peptidic nanofibrils in the co-culture. The bright yellow fluorescence, resulted from the nanofibrils of f-DTP, mainly localizes on HeLa-GFP cells, and only few fainted yellow dots in HS-5 cells (Figure 1h and Supplementary Figure 2d). Collectively, these results confirm that ALPs, as ectoenzymes overexpressed on cancer cells, generate DTP nanofibrils *in situ* for selectively killing cancer cells without harming normal cells.

### ALP-generated nanofibrils pleiotropically activate extrinsic death signaling

**Figure 2:**
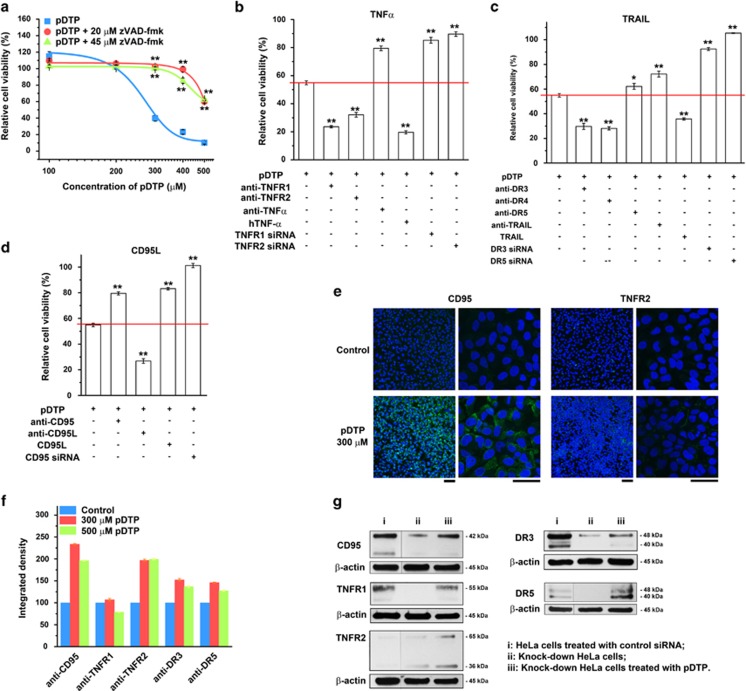
Pericellular D-peptide nanofibrils act as multifaceted inducer of apoptosis. (**a**) Relative cell viability of HeLa cells treated with different concentrations of pDTP and 20 or 45 *μ*M of pan-caspase inhibitor zVAD-fmk for 48 h. The initial number of cells is 1.0 × 10^4^/well. *n*=3. Relative cell viability of HeLa cells treated with 300 *μ*M pDTP and (**b**) TNF-*α*-related mAbs; (**c**) TRAIL-related mAbs; or (**d**) CD95L-related mAbs for 48 h. Data are shown as mean±S.D. *P<0.05, **P<0.01 by Student's *t*-test. (**e**) The overlaid confocal fluorescent microscope images (× 20 dry (left) or × 100 oil (right) objective lens) show the clustering of the cell death receptors. Cells are treated with death receptor antibodies (e.g., anti-CD95 or anti-TNFR2 mAb) and growth medium (control) or 300 *μ*M of pDTP in growth medium. Blue indicates all the live cells stained by Hoechst 33342 at the concentration of 1 *μ*g/ml; and green indicates the secondary antibodies. The initial number of cells is 1.0 × 10^5^/well. The scale bar is 100 *μ*m for low magnification (left) and 50 *μ*m for high magnification (right). (**f**) The quantification of integrated density of fluorescence of HeLa cells treated with 300 *μ*M or 500 *μ*M of pDTP in growth medium and the cell death receptor mAbs. (**g**) Western blot shows the expression of death receptors (i.e., CD95, TNFR1, TNFR2, DR3, and DR5) in HeLa cells treated with control siRNA, knock-down HeLa cells, or knock-down HeLa cells treated with 300 *μ*M of pDTP for 24 h

We focus on extrinsic cell death signaling because previous results suggest the ALP-generated DTP nanofibrils inducing mitochondrial independent cell death.^7^ Co-incubating zVAD-fmk^34^ (a pan-caspase inhibitor) with pDTP significantly reduces the cell death (Figure 2a), indicating that DTP nanofibrils mainly induce apoptosis. We co-incubate pDTP with extrinsic cell death ligands and monoclonal antibodies (mAbs) of key extrinsic cell death signaling molecules, respectively. While anti-TNF-*α* considerably protects cells, anti-TNFR1 or anti-TNFR2 leads to more cell death (Figure 2b and Supplementary Figures 3a–c). hTNF-*α* also causes more cell death without the use of cycloheximide^35^ (Supplementary Figure 3d). These results indicate that DTP nanofibrils enhance interactions between the autocrine TNF-*α*^36^ and its cognate receptors, likely by presenting TNF-*α* in a juxtacrine manner.^37^ siRNA knock-down^38^ of TNFR1 or TNFR2 in HeLa cells decreases the cytotoxicity of pDTP (Figure 2b and Supplementary Figure 3l), agreeing with the juxtacrine presentation of TNF-*α* (as well as anti-TNFR1 or anti-TNFR2) by DTP nanofibrils (Figure 1a). While anti-DR5 exhibits little effect, anti-DR3, anti-DR4, or TRAIL slightly promotes cell death (Figure 2c and Supplementary Figures 3e, 3f, and 3h). Anti-TRAIL only slightly decreases the cytotoxicity of DTP nanofibrils (Supplementary Figure 3g), but knock-down of DR3 or DR5 considerably rescues the cells (Supplementary Figure 3l). These results suggest that DTP nanofibrils, besides interacting with TRAILRs via the juxtacrine presentation of TRAIL, directly interact with TRAILRs. Either anti-CD95 or CD95L improves the cell survival, anti-CD95L results in more cell death (Figure 2d and Supplementary Figure 3i–k), and knock-down of CD95 decreases the cytotoxicity of pDTP (Supplementary Figure 3l). These results indicate that DTP nanofibrils directly interact with CD95 to cause apoptosis (Figure 1a). Although the siRNA knock down of two cell death receptors simultaneously (e.g., CD95 and TNFR1, CD95 and TNFR2, or TNFR1 and TNFR2) is less effective than the knock down of one death receptor, it still protects HeLa cells from DTP nanofibrils (Supplementary Figures 3m–p) at certain extent.

After the addition of pDTP, primary mAbs of death receptors, and the fluorescent secondary mAb, cell imaging reveals significantly increased clustering of CD95 and moderately induced aggregation of TNFR2 and DR3 (Figure 2e and Supplementary Figures 4a–c), agreeing with clustering the death receptors as a key feature of apoptosis signaling.^39^ Together with the integrated intensity of fluorescence (Figure 2f), these results further support the mechanism in Figure 1a. As the controls, anti-TNFR1 or anti-TNFR2 co-incubating with DTP in HeLa culture (Supplementary Figure 5a) or with pDTP in HS-5 culture (Supplementary Figure 5b) exhibits little effect on cell viabilities, confirming that ALP *in situ* generating DTP nanofibrils is the critical process for selective inhibition of cancer cells (Figure 1a). Moreover, The knock-downed death receptors (i.e., TNFR2, CD95, or DR5) re-emerge (Figure 2g) after incubating the siRNA-treated HeLa cells with pDTP, suggesting that DTP nanofibrils interact with other unknock-downed death receptors to induce the re-emergence, likely via molecular crosstalk between cell death and survival signaling.^40^

**Figure 3:**
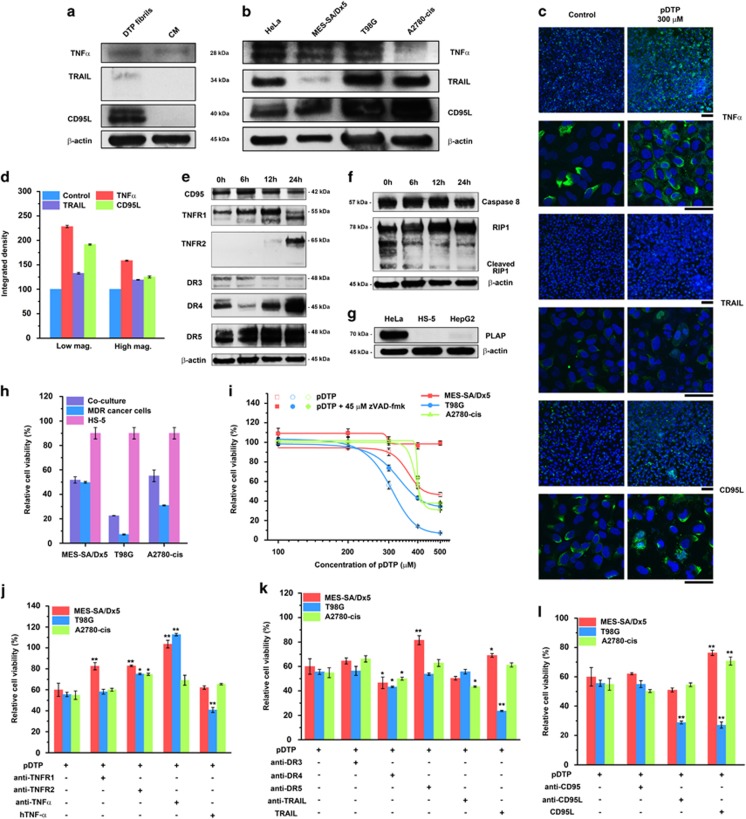
Pericellular D-peptide nanofibrils selectively inhibit cancer cells in co-culture via presenting autocrine death ligands, and act as a multifaceted death signal of the apoptosis of cancer cells. Western blot shows the difference of death ligands (**a**) in pericellular DTP nanofibrils and conditioned medium (CM) with HeLa cells; (**b**) in four kinds of cell lysates (i.e., HeLa, MES-SA/Dx5, T98G, A2780-cis). (**c**) Fluorescent images show the expression of cell death ligands. Blue indicates all the live cells stained by Hoechst 33342; and green indicates the secondary antibodies. The initial number of cells is 1.0 × 10^5^/well. The scale bar is 100 *μ*m for low magnification (up) and 50 *μ*m for high magnification (down). (**d**) The quantification of integrated density of fluorescence of HeLa cells showed in **c**. Western blot shows change of relative amount of (**e**) cell death receptors; (**f**) down-stream proteins over time in HeLa cells treated by 300 *μ*M of pDTP. (**g**) Western blot shows the difference of PLAP expressed on membrane of three kinds of cells (i.e., HeLa, HS-5, HepG2). (**h**) Relative cell viability of the co-cultured cells (HS-5 and MDR cancer cells) incubated with pDTP in DMEM for 48 h. The initial number of cells is 1.0 × 10^4^/well (e.g., 1.0 × 10^4^ MDR or HS-5 cells, or mixture of 5.0 × 10^3^ MDR cells and 5.0 × 10^3^ HS-5 cells). (**i**) Relative cell viability of MDR cells treated with pDTP and zVAD-fmk for 48 h. Relative cell viability of MDR cells treated with 400 or 500 *μ*M pDTP and (**j**) TNF-*α*-related mAbs; (**k**) TRAIL-related mAbs; or (**l**) CD95L-related mAbs for 48 h. The initial number of cells is 1.0 × 10^4^/well. *n*=3. Data are shown as mean±S.D. *P<0.05, **P<0.01 by Student's *t*-test

We compare the amounts of secreted extrinsic cell death ligands trapped in the pericellular DTP nanofibrils and those in the conditioned medium of HeLa cells and find that DTP nanofibrils retain little TRAIL, a moderate level of TNF-*α*, and CD95L significantly (Figure 3a). Although the cell lysate of HeLa contains TNF-*α*, TRAIL, and CD95L (Figure 3b), only CD95L exhibits the considerably high amount in the pericellular DTP nanofibrils, agreeing with that cancer cells, under stress, increase the secretion of CD95L. This result further supports that DTP nanofibrils present the autocrine death ligands (Figure 3b). In addition, the western blot reveals that pDTP exhibits little effect on the expression of TNF-*α* or TRAIL while moderately increases the expression of CD95L (Supplementary Figure 5h), suggesting that the pericellular DTP nanofibrils, besides increasing autocrine CD95L of HeLa cells, mainly retain the autocrine death ligands near the cell surface to interact with the death receptors. However, it is also possible that DTP nanofibrils possess slightly higher affinity to CD95L and CD95 than to TNF-*α* and TRAIL. Nevertheless, the fluorescent imaging of HeLa cells treated with pDTP, primary mAbs of the ligands, and the fluorescent secondary mAb (Figure 3c), together with quantification of the fluorescence (Figure 3d), indicates that the pericellular DTP nanofibrils increase the clustering of death ligands (e.g., TNF-*α*, CD95L), which is consistent with that DTP nanofibrils present the autocrine proapoptotic ligands to their cognate receptors in a juxtacrine manner.

To further confirm that DTP nanofibrils are able to present the death ligands, we test the effect of pDTP on CD95L-KO HeyA8 cells (Supplementary Figure 5c). pDTP only exhibits weak cytotoxicity to CD95L-KO HeyA8 cells at 48 h. But the addition of CD95L together with pDTP significantly increases the CD95L-KO HeyA8 cell death. This result supports the notion that DTP nanofibrils present CD95L to induce apoptosis.

For the pDTP-treated HeLa cells, Western blot reveals that, while the constitutive expression of TNFR1 increases initially and then decreases moderately, the inductive expression of TNFR2, especially the glycosylated form (75 kD),^41^ significantly increases over time. While the expressions of CD95 and DR3 decrease slightly, expression of DR4 increases remarkably, and expression of DR5 increases moderately (Figure 3e). None of these changes of TNFR1 and TNFR2 occur in the controls (Supplementary Figures 5d–g), confirming that the ALP-generated DTP nanofibrils result in those changes. Western blot of the down-stream proteins (e.g., caspase 8 or RIP1) shows that pDTP slightly decreases the expression of caspase 8 and the cleaved RIP1 while increases expression of RIP1 (Figure 3f), agreeing with that DTP nanofibrils mainly result in apoptosis. In the case of HeLa cells, we find that cleaved caspase 8 appears at 24 h, cleaved caspase 3 emerges around 36 h, and cleaved PARP also arises in small amount at 12 h and is significantly produced around 24 h. These results (Supplementary Figure 5i) confirm DTP nanofibrils activate caspases for apoptosis of HeLa cells. In addition, HeLa cells overexpress PLAP on membrane, while HS-5 and HepG2 cells barely express PLAP (Figure 3g). This fact, together with the cell viabilities of these three cells treated by pDTP (Supplementary Table 1), suggests that pDTP acts as the substrate of ALP (e.g., PLAP) on HeLa cells to form the nanofibrils that induce apoptosis. These results demonstrate DTP nanofibrils as multifaceted inducers of apoptosis by pleiotropically interacting with extrinsic cell death ligands and receptors.

### ALP-generated nanofibrils selectively kill MDR cancer cells

As shown by western blot (Supplementary Figure 6a), several MDR cancer cells (e.g., MES-SA/Dx5, T98G, and A2780-cis) also overexpress ALPs. Besides overexpressing germ cell alkaline phosphatases, MES-SA/Dx5, T98G, and A2780cis overexpress tissue non-specific alkaline phosphatases. So we evaluate the responses of these three MDR cells to pDTP, including in their co-cultures with HS-5, and examine the corresponding cell death mechanisms. pDTP selectively induces apoptosis of those MDR cancer cells while remains innocuous to HS-5 cells in their co-cultures (Figure 3h and Supplementary Figure 6b), agreeing with the viability results in their separated cultures (Supplementary Table 1). zVAD-fmk, co-incubated with pDTP, almost completely rescues MES-SA/Dx5, significantly protects T98G, and moderately reduces the death of A2780-cis (Figure 3i), indicating that DTP nanofibrils induce apoptosis of those cells, albeit at different extent. hTNF-*α* leads to more death of T98G than those of MES-SA/Dx5 and A2780-cis; anti-TNF-*α* significantly protects MES-SA/Dx5 and T98G, anti-TNFR1 rescues MES-SA/Dx5; anti-TNFR2 moderately protects MES-SA/Dx5, T98G, and A2780-cis (Figure 3j and Supplementary Figure 6c). TRAIL results in more death of T98G, but offers slight protection to MES-SA/Dx5 and A2780-cis; anti-TRAIL slightly promotes the death of MES-SA/Dx5 and A2780-cis; anti-DR3 slightly protects MES-SA/Dx5; anti-DR4 slightly increases cytotoxicity of all the three cells; anti-DR5 mainly protects MES-SA/Dx5 (Figure 3k and Supplementary Figure 6d). CD95L also causes more death of T98G, but offers considerable protection to MES-SA/Dx5 and A2780-cis; anti-CD95L only leads more death of T98G; anti-CD95 hardly affects MES-SA/Dx5, T98G, and A2780-cis cells (Figure 3l and Supplementary Figure 6e). Both anti-CD95L and CD95L increase cell death of T98G, indicating that the pericellular DTP nanofibrils may directly interact with CD95 receptors as well as present autocrine CD95L of T98G cells. These apparently paradoxical results reflect the complexity and versatility of the EISA process and molecular nanofibrils. These results indicate that DTP nanofibrils directly interact with CD95 and DR5 or present TNF-*α* to induce death of MES-SA/Dx5 and A2780-cis, but mainly present TNF-*α*, TRAIL, and CD95L to their cognate receptors inducing apoptosis of T98G cells.

Table 1 summarizes the role of the receptor/ligand pairs in different cell lines contributed to the apoptosis induced by pDTP. Unlike HeLa cells that express the three extrinsic cell death receptors, the other cell lines lack the expression of at least one death receptor/ligand pair. The absence of the death receptors may contribute to the resistance of these tumor cells to the corresponding death ligands.

**Figure 4:**
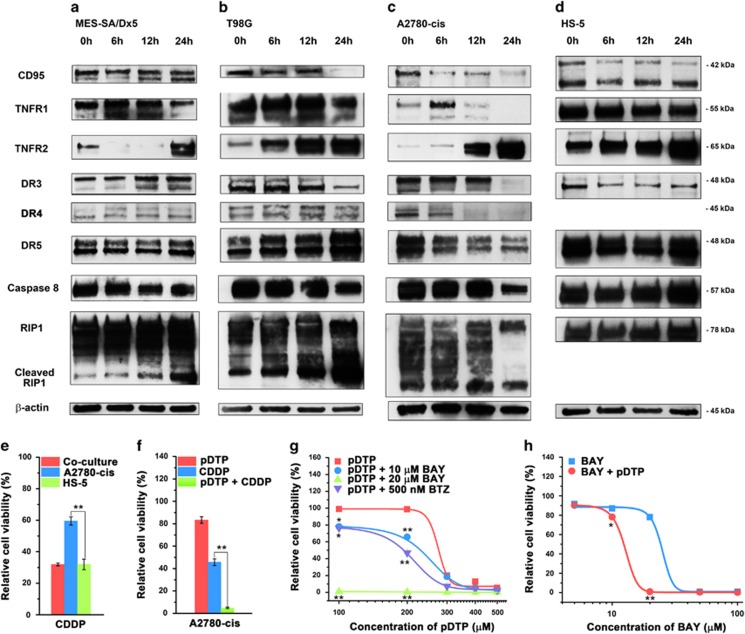
Pericellular D-peptide nanofibrils affect the expression of death receptors and selectively inhibit MDR cancer cells for combination therapy. Western blot shows change of relative amount of cell death receptors and two down-stream proteins over time in (**a**) MES-SA/Dx5, (**b**) T98G, (**c**) A2780-cis, or (**d**) HS-5 cells treated by pDTP. (**e**) Relative cell viability of the co-culture of A2780-cis and HS-5, A2780-cis, and HS-5 cells incubated with cisplatin (CDDP) at the concentration of 6 *μ*g/ml in DMEM. (**f**) Relative cell viability of A2780-cis cells incubated with 216 *μ*g/ml pDTP, 15 *μ*g/ml CDDP, or the mixture of 216 *μ*g/ml pDTP and 15 *μ*g/ml CDDP for 24 h. Cells are pretreated with CDDP for 12 h. Relative cell viability of HeLa cells incubated with (**g**) pDTP of different concentrations and 10 or 20 *μ*M BAY 11-7085 or 500 nM BTZ; (**h**) BAY of different concentrations and 100 *μ*M pDTP. The incubation time is 48 h. The initial number of cells is 1.0 × 10^4^/well. *n*=3. Data are shown as mean±S.D. *P<0.05, **P<0.01 by Student's *t*-test

Western blot results show that the increasing inductive expression of TNFR2 and the up-and down-expression of TNFR1 over time (Figures 4a–c) are the common trends for these MDR cells incubated with pDTP. DR3 slightly increases on MEA-SA/Dx5, but decreases on T98G and varies on A2780-cis. DR5 exhibits little change on MES-SA/Dx5, increases on T98G, and decreases on A2780-cis (Figures 4a–c). These results indicate that the molecular crosstalk induced by DTP nanofibrils depends on cell types, partly due to the cell specific expressions of death ligands (Figures 3b). Meanwhile, pDTP results in changing the expression of caspase 8 and the fragmentation of RIP1 in these cell lines (Figures 4a–c), further supporting the cell death largely via apoptosis. For HS-5 cells, pDTP results in little increase of TNFR2 and hardly affects expressions of other signaling molecules (e.g., TNFR1, caspase 8, or RIP1) (Figure 4d and Supplementary Figure 7a). HepG2 cells, a type of cancer cells being viable upon the pDTP treatment (Supplementary Figure 7b), exhibit decreasing the expression of TNFR2 (Supplementary Figures. 7c and d). These results establish ALP-generated DTP nanofibrils pleiotropically activating extrinsic cell death signaling as a general mechanism for triggering apoptosis of ALP-overexpressing cancer cells.

### Combination of nanofibrils with CDDP or NF-*ĸ*B inhibitors against MDR cancer cells

We compare the selectivity of cis-diamminedichloroplatinum(II) (CDDP),^27^ a clinical drug, with that of pDTP, and combine pDTP with CDDP or NF-*ĸ*B inhibitors (e.g., BTZ^28^ or BAY(11-7085)^29^) for killing cancer cells. CDDP, unlike pDTP, inhibits cells with little selectivity (Figure 4e and Supplementary Figure 7e), even killing more HS-5 than to A2780-cis cells. pDTP turns the IC_50_ value of CDDP against A2780-cis cells to an IC_95_ value (Figure 4f and Supplementary Figures 7f and g), promising molecular nanofibrils for CDDP-based combination therapy.^15^ The presence of NF-*ĸ*B inhibitors (e.g., BTZ or BAY) considerably decreases the IC_50_ value of pDTP (Figures 4g and h and Supplementary Figures 7h and i) and even induces great cytotoxicity with lower concentration of pDTP (i.e., 3.6 *μ*g/ml, Supplementary Figure 7j) or with less incubation time (i.e., 2 h, Supplementary Figure 7k). These results agree with that DTP nanofibrils activate extrinsic cell death signaling and suggest the potential use of pDTP to enhance the efficacy and selectivity of NF-*k*B inhibitors against cancer cells of solid tumors.

### Peritumoral test in a murine model

**Figure 5:**
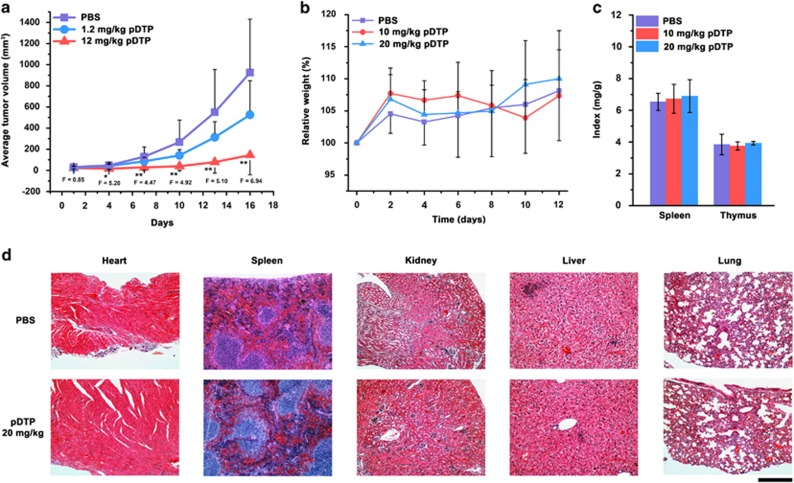
Pericellular D-peptide nanofibrils selectively inhibit MES-SA/Dx5 tumor on nu/nu mice. (**a**) Tumor progression curves of mice bearing MES-SA/Dx5 tumors. In all, 0.1 ml of pDTP at 1.2 mg/kg, 12 mg/kg, or just PBS buffer as control was injected subcutaneously and peritumorally in every 3 days (five doses, starting day 1). Data are shown as mean±S.D. (each group contains six mice). *P<0.05, **P<0.01 by Student's *t*-test. *F* values of every 3 days by Anova statistic with the critical value *F*=3.68. The change of the (**b**) body weights or (**c**) spleen or thymus index of nu/nu mice during the treatment. In all, 0.1 ml of pDTP at 10 mg/ml, 20 mg/ml, or just PBS buffer as control was injected intravenously (starting day 0), and then the body weights or spleen or thymus index of mice were tested. (**d**) Hematoxylin and eosin (H&E) staining images of heart, spleen, kidney, liver, or lung from the nu/nu mice treated with 0.1 ml of pDTP at 20 mg/ml, or just PBS buffer as control by intravenous injection. The scale bar is 100 *μ*m

We measure the tumor growth in xenograft tumor mice model with MES-SA/Dx5 cells (Figure 5a and Supplementary Figure 8a) and find that the peritumoral treatment of pDTP significantly slows down the progression of tumor (i.e., seven times reduction comparing with the control group). The body weights of treated mice are comparable with those in the control group (Supplementary Figure 8b), and the skins covering and around tumor appear to be normal (i.e., without ulceration or sclerosis, Supplementary Figure 8c) on every mice treated with pDTP, agreeing with DTP as innocuous monomers.^7^ In addition, pDTP hardly forms DTP nanofibrils in human serum (Supplementary Figures 8d and e), suggesting low toxicity to *in vivo*. Thus, we conduct *in vivo* toxicity test in mice. The intravenous injection of pDTP has little adverse effect on mice, without changing body weight, spleen, thymus, and blood conditions (Figures 5b and c and Supplementary Figures 9a–e). H and E staining indicates that major organs (e.g., heart, spleen, kidney, liver, and lung) of the injected mice are normal (Figure 5d and Supplementary Figure 9f), further confirming low *in vivo* toxicity of pDTP.

### ALP generating nanofibrils as a multitargeting process for apoptosis

The goal of cancer therapy is to kill cancer cells without harming normal cells. Although the generic differences between cancer and normal cells are rare, recent tissue-based map of human proteome^42^ indicates that overexpression of ALPs, one of the earliest tumor markers identified,^31^ is a generic difference between cancer and normal cells. Moreover, it is common for late stage cancers overexpress ALPs.^43^ However, ALP is ‘undruggable'. Thus, the use of EISA as a process to generate nanofibrils as multifaceted, localized inducers for cell death signaling may be particularly valuable to address the problem of MDR in cancer therapy.

DTP combined with anti-TNFR1/2 mAbs has no effect on HeLa cells (Supplementary Figure 5a) since the assembly of DTP hardly localizes on cell surface without ALP catalysis. Anti-TNFR1 or anti-TNFR2 makes little difference on the viability of HS-5 treated with pDTP (Supplementary Figure 5b) because HS-5 expresses little ALP on cell surface (Figure 3g). In addition, unlike the case of HeLa cells treated with pDTP, western blot analysis of HeLa cells incubated with DTP itself, pDTP and L-Phe, or pDTP and ALP shows little change of the death receptors or down-stream proteins over time (i.e., little effect on the expression of TNFR1 and TNFR2). These results further confirm that the formation of DTP nanofibrils, a multiple step molecular process catalyzed by ALP *in situ*, is necessary for the selective inhibition of the cancer cells.

It is known that the co-incubation of a NF-*ĸ*B inhibitor (e.g., cycloheximide) is necessary for TNF-*α*-induced apoptosis,^35^ an observation has led to the recognition of the difference between paracrine and juxtacrine signaling of TNF-*α*.^44^ This work reports that hTNF-*α* combined with pDTP induces cell death without NF-*ĸ*B inhibitors (Figure 2b), a strong evidence of DTP nanofibrils promoting the juxtacrine signaling. Because PLAP catalyzes the formation of DTP nanofibrils, the inclusion of exogenous hTNF-*α* in the nanofibrils for juxtacrine signaling can occur during the self-assembly of DTP, which agrees with that more cells are dead upon the addition of hTNF-*α*. Moreover, the addition of anti-TNFR1 and anti-TNFR2 causes more death of HeLa cells incubated with pDTP, not only agreeing with that antibodies to TNF receptor can have TNF-like activity,^45^ but also suggesting that nanofibrils may crosslink/aggregate the antibody to convert an otherwise neutralizing antibody to an agonistic antibody. In addition, the combination of pDTP and NF-*ĸ*B inhibitors against cancer cells shows that pDTP enhances the efficacy of NF-*ĸ*B inhibitors (Figures 4g and h). NF-*ĸ*B inhibitors also increase the inhibition ability of pDTP against cancer cells. These results further demonstrate DTP nanofibrils to bias NF-*ĸ*B pathway for cell death, suggesting that it might be worthwhile to combine pDTP with other NF-*ĸ*B inhibitors for further elucidating the detailed mechanisms depending on specificity of NF-*ĸ*B transcription factors.

Interestingly, the knock-down of either TNF-*α*, TRAIL, or CD95L receptors all rescues cell death ~50%, strongly suggesting crosstalk between all death receptor pathways at the level of receptor clustering and/or engagement. In addition, western blot analysis shows that DTP nanofibrils cause the re-emergence of death receptors (especially TNFR1, TNFR2, and DR5) on siRNA knock-down cells (Figure 2g). This result indicates that, even if one kind of death receptors is knocked down, DTP nanofibrils can affect or interact with other receptors, regulate their down-stream signaling, and induce the re-emergence of silenced receptors, implying that the nanofibrils may counter cancer drug resistance^13^ due to downregulation of a death receptor. As the first mechanistic study on multitargeting by enzyme-triggered nanofibrils to result in apoptosis of cancer cells, this work also implies that the use of locally generated nanofibrils to present proapoptotic ligands (e.g., TNF-*α*, CD95L, and/or TRAIL) in a juxtacrine mode may lead a way to bringing back the early promises of proapoptotic ligands for cancer therapy.

## Materials and methods

### Transmission electron microscopy (TEM)

Before placing sample solution/gels on the grid (3 *μ*l, sufficient to cover the grid surface), we glow discharge the carbon-coated grids to increase their hydrophilicity. About 10 s later, we place three large drops of ddH_2_O on parafilm to let the grid touch the water drops with the sample-loaded surface facing the parafilm, and then tilt the grid and gently absorb water from the edge of the grid using a filter paper. Immediately after rinsing, we place three large drops of uranyl acetate (UA) staining solution on parafilm to let the grid touch the staining solution drops with the sample-loaded surface facing the parafilm, and then tilt the grid and gently absorb the stain solutions from the edge of the grid using a filter paper. After drying the grid in air and it is ready for EM imaging, we examine the grid as soon as possible.^46^ Solutions and supplies: 2.0% (w/v) UA (prepare by dissolving 200 mg UA in 10 ml of ddH_2_O. Add water to the tube containing UA, cover tube with foil and rotate in cold room for several hours till fully dissolved. Filter through a 0.22-*μ*m filter that has be pre-rinsed well with ddH_2_O. Filtered stain stored at 4 °C in a foil-wrapped tube can be used for >1 year); filter strips (prepared by cutting Whatman 1 filter paper into small slivers); grids (400 mesh copper grids coated with continuous thick carbon film ~35 nm in thickness, purchased from Pacific Grid Tech. Co). We prepare samples by adding ALP (5 U/ml) to the solution of pDTP at concentration of 362 *μ*g/ml.

### Cells culture

HeLa, T98G, HS-5, HepG2 cells are purchased from the American Type Culture Collection (ATCC, Manassas, VA, USA), MES-SA/Dx5 and A2780-cis cells from Sigma-Aldrich Co. (St. Louis, MO, USA), and HeLa-GFP cell from Cell Biolabs, Inc. (San Diego, CA, USA) HeLa, T98G, HeLa-GFP, HepG2 cells are propagated in minimum essential media (MEM, Invitrogen Life Technologies); HS-5 cell is cultured in Dulbecco's modified Eagle's medium (DMEM, Invitrogen Life Technologies, Waltham, MA, USA); MES-SA/Dx5 cell is propagated in McCoy's 5A (Invitrogen Life Technologies); A2780-cis cell is propagated in RPMI-1640 medium (ATCC). A2780-cis is supplemented with 10% FBS, 2 mM glutamine, and 1 *μ*M cisplatin is necessary every 2–3 passages. The rest of the above cells are supplemented with 10% fetal bovine serum (FBS, Invitrogen Life Technologies) and 1% of antibiotics (100 U/ml penicillin and 100 *μ*g/ml streptomycin, Invitrogen Life Technologies). For the co-culture of cancer and normal cells, the cancer cells (HeLa-GFP, MES-SA/Dx5, T98G, A2780-cis) and normal cells (HS-5) are cultured in DMEM supplemented with 10% of FBS and 1% of antibiotics. All cells are incubated in a fully humidified incubator containing 5% CO_2_ at 37 °C. For the co-culture of two different cell lines, we mix same amount of two cells and incubate them in DMEM.

### Cell viability assay

Cells in exponential growth phase are seeded in a 96-well plate at a density of 1 × 10^4^ cells/well. The cells are allowed to attach to the wells for 4 h at 37 °C, 5% CO_2_. The culture medium is removed and 100 *μ*l culture medium containing compounds (immediately diluted from fresh prepared stock solution of 10 mM) at gradient concentrations (0 *μ*M as the control) is placed into each well. After culturing at 37 °C, 5% CO_2_ for 24, 48, 72 h, each well is added by 10 *μ*l of 5 mg/ml MTT ((3-(4, 5-dimethylthiazol-2-yl)-2, 5-diphenyltetrazolium bromide),^47^ and the plated cells are incubate at dark for 4 h. One-hundred microliters 10% SDS with 0.01 M HCl is added to each well to stop the reduction reaction and to dissolve the purple. After incubation of the cells at 37 °C for overnight, the OD at 595 nm of the solution is measured in a microplate reader (DTX 880 multimode detector). Data represent the mean±standard deviation of three independent experiments.

### Live cell imaging

HeLa-GFP and HS-5 cells in exponential growth phase are seeded in glass bottomed culture chamber at 1 × 10^5^ cells/well (5 × 10^4^ HeLa-GFP and 5 × 10^4^ HS-5 cells per well). The cells are allowed for attachment for 24 h at 37 °C, 5% CO_2_. The culture medium is removed, and new culture medium containing pDTP at 216 or 362 *μ*g/ml or f-pDTP at 493 *μ*g/ml is added. After incubation for certain time (e.g., 24 h for pDTP or 4 h for f-pDTP), cells are stained with 1.0 *μ*g/ml Hochst 33342 for 5 min at 37 °C in dark. After that, cells are rinsed three times by PBS buffer, and then kept in PBS buffer for imaging.

### Cell imaging with antibody staining

After incubating 1 × 10^5^ HeLa cells per glass bottomed culture chamber for 24 h at 37 °C, 5% CO_2_, the cells are treated with 216 or 362 *μ*g/ml of pDTP for 24 h. After three times rinse by PBS buffer, cells are 4% formaldehyde fixed (10 min) and then incubated in 1% BSA/10% normal goat serum/0.3 M glycine in 0.1% PBS-Tween for 1 h to permeabilize the cells and block non-specific protein–protein interactions. The cells are then incubated with the primary antibodies (e.g., anti-CD95, anti-TNFR1, anti-TNFR2, anti-DR3, anti-DR5 mAbs) overnight at +4 °C. The secondary antibody (green) is Alexa Fluor 488 goat anti-rabbit IgG (H+L) (abcam) used at 2 *μ*g/ml for 1 h. Hochst 33342 is used to stain the cell nuclei (blue) at a concentration of 1.0 *μ*g/ml.^48^ The fluorescent intensity in each image is analyzed using ImageJ.^49^

### Knock-down HeLa cells

In a 3 cm petri dish, we seed 2 × 10^5^ HeLa cells in 2 ml antibiotic-free normal growth medium supplemented with FBS and incubate the cells at 37 °C in a CO_2_ incubator for 24 h to make cells 60–80% confluent. We prepare two solutions, siRNA duplex solution (diluting 32 *μ*l siRNA duplex (Santa Cruz Biotechnology, Inc., Dallas, TX, USA) into 100 *μ*l siRNA transfection medium (Santa Cruz Biotechnology, Inc.)) and the dilute transfection reagent solution (dilute 6 or 32 *μ*l siRNA transfection reagent (Santa Cruz Biotechnology, Inc.) into 100 *μ*l siRNA transfection medium), and then mix gently the two solutions by pipetting the solution up and down and incubate the mixture 45 min at room temperature. After washing the cells once with 2 ml of siRNA transfection medium, we immediately dilute the siRNA duplex mixture with 0.8 ml transfection medium and overlay the mixture onto the washed cells. After incubating the cells 7–12 h (depending on the cell conditions) at 37 °C in a CO_2_ incubator, 1 ml of normal growth medium containing two times the normal serum and antibiotics concentration (2 × normal growth medium) is added without removing the transfection mixture (if toxicity is still a problem, we would remove the transfection mixture and replace with 1 × normal growth medium). In all, 18–24 h later, we aspirate the medium and replace with fresh 1 × normal growth medium, and then the cells are ready for western blot or cell viability assay after 72 more hours.^50^

### Time-dependent western blot

Four of 10 cm petri dishes seeded 2 × 10^6^ of HeLa, MES-SA/Dx5, T98G, A2780-cis, HS-5, or HepG2 cells onto each dish are prepared for the time-dependent western blotting experiment. After overnight incubation, we randomly choose three of them to be incubated with medium containing 300 *μ*M of pDTP, or just fresh medium. The other dish of cells is washed with PBS buffer for three times and scrapped from petri dish after adding 500 *μ*l cell lysis buffers, which represents the cell lysates at 0 h. Similarly, we collect one dish of treated cell lysates at 6 h, 12 h, 24 h, respectively, by using the same method. The collected cell lysates are mixed with 5 *μ*l of 100 × proteinase inhibitor cocktail and then snap-frozen and thawed for three cycles to lyse the cells. The cell lysates are clarified by centrifugation at 12 000 × *g* for 20 min at 4 °C to remove the whole cells, nuclei, and large mitochondria. After quantification of proteins in cell lysates by using the Bradford reagent, the same amount of proteins for different time is mixed with the same volume of 2 × Laemmli buffer before SDS-PAGE. After finishing the gel electrophoresis and transferring the proteins on the gels to the PVDF membranes for overnight, the membranes are incubated with primary antibodies (1:1000, all antibodies were obtained from Cell Signaling Technology, Danvers, MA, USA) for overnight. The membranes are then incubated with secondary antibodies (1:2000) for 1 h, followed by the reaction with ECL solutions for 10 min and the final detection of proteins.^51^

### Western blot of L-Phe or ALP co-incubated cells

Similar to the protocol of time dependent western blot, 2 × 10^6^ of HeLa cells are seeded onto each 10 cm petri dish initially. After overnight incubation, three random dishes would be incubated with medium containing 300 *μ*M of pDTP and 5 mM of L-phenylalanine (L-Phe). Another three random dishes would be incubated with medium containing 300 *μ*M of pDTP and 10 U/ml of alkaline phosphatase (ALP, Biomatik Corporation, Wilmington, DE, USA). Similarly, we collect the cell lysates at 0, 6, 12, and 24 h, respectively. After quantification of proteins in cell lysates by using the Bradford reagent, the same amount of proteins for different time is used for SDS-PAGE and western blotting.

### *In vivo* tumor model with MES-SA/Dx5

The animal care facilities and programs of the Foster Animal Research Facility in Brandeis University meet the requirements of the law and NIH regulations. The animals are subjected to regular veterinary care on a routine basis. The animal experiments described in this study are performed according to the *Guide for the Care and Use of Laboratory Animals* published by the NIH. The nu/nu mice are purchased from Charles River Laboratories (Wilmington, MA). MES-SA/Dx5 cells are collected in exponential growth phase. In all, 100 *μ*l of MES-SA/Dx5 cells suspension in complete culture medium at 5 × 10^7^ cell/ml is subcutaneously injected into the right flank of nu/nu mice.^52^ After 14 days of inoculation, mice bearing MES-SA/Dx5 tumor at average volume of 30 mm^3^ are randomly divided into three groups. Subcutaneous peritumoral injections are performed every 3 days for five doses. One group is treated with 100 *μ*l of pDTP at 3.6 mg/ml in PBS buffer; the other group is treated with 100 *μ*l of pDTP at 0.36 mg/ml in PBS buffer; control group is treated with 100 *μ*l PBS buffer. Based on the average body weight of the mice (22 g) and the molar mass of pDTP (723.23 g/mol), the dosages in gram per kilogram of body weight of pDTP on the mice were calculated as follow: 100 *μ*l × 3.6 mg/ml × 723.23 g/mol/22 g=12 mg/kg; 100 *μ*l × 0.36 mg/ml × 723.23 g/mol/22 g=1.2 mg/kg. The number of mice in each group is six. The statistical significance in relative tumor size between the control group and the group treated by 12 mg/kg of pDTP is examined by Student's *t*-test and Anova statistical analysis.

### *In vivo* assessment of pDTP by intravenous injection

Similar to the protocol mentioned in nu/nu mice, we randomly divide mice into four groups: 10, 20, 50 mg/kg pDTP, and control group (PBS buffer). For each mouse, we would inject pDTP or PBS buffer every 3 days intravenously, and then measure the body weight of mice, organ index, or parameters of enzymes or proteins. In addition, we take H and E staining images of heart, spleen, kidney, liver, and lung from the nu/nu mice. The number of mice in each group is six.

## Figures and Tables

**Table 1:** The summary of the receptor/ligand pairs required for pDTP toxicity in different cell lines (↑ indicates increasing the cytotoxicity;↓indicates decreasing the cytotoxicity; − indicates little effect)

**Cells**	**HeLa**	**MES-SA/Dx5**	**T98G**	**A2780-cis**
*mAbs*				
Anti-TNFR1	↑	↓	−	−
Anti-TNFR2	↑	↓	↓	↓
Anti-TNF-*α*	↓	↓	↓	−
TNF-*α*	↑	−	↑	−
Anti-DR3	↑	−	−	−
Anti-DR4	↑	↑	↑	−
Anti-DR5	−	↓	−	−
Anti-TRAIL	↓	−	−	↑
TRAIL	↑	−	↑	−
Anti-CD95	↓	−	−	−
Anti-CD95L	↑	−	↑	−
CD95L	↓	↓	↑	↓
