# Supplementary material for: In situ generated D-peptidic nanofibrils as multifaceted apoptotic inducers to target cancer cells
Source: Cell Death Dis. 2017 Feb 16;8(2):e2614–. doi: 10.1038/cddis.2016.466 (PMC5386457; doi:10.1038/cddis.2016.466)
Supplement: Supplementary Information [file cddis2016466x1.docx]

***In Situ* Generated D‐Peptidic Nanofibrils as Multifaceted Apoptotic Inducers to Target Cancer Cells**

Xuewen Du^1^, Jie Zhou^1^, Huainin Wang^1^, Junfeng Shi^1^, Yi Kuang^1^, Wu Zeng^2^, Zhimou Yang^3^, and Bing Xu^1,^*

^1^Department of Chemistry, Brandeis University, Waltham, MA 02454, USA. ^2^Schneider Institutes for Health Policy, Brandeis University, Waltham, MA 02454, USA. ^3^College of Life Sciences, Nankai University, Tianjin, 300071, China.

*E-mail: [bxu@brandeis.edu](mailto:bxu@brandeis.edu)


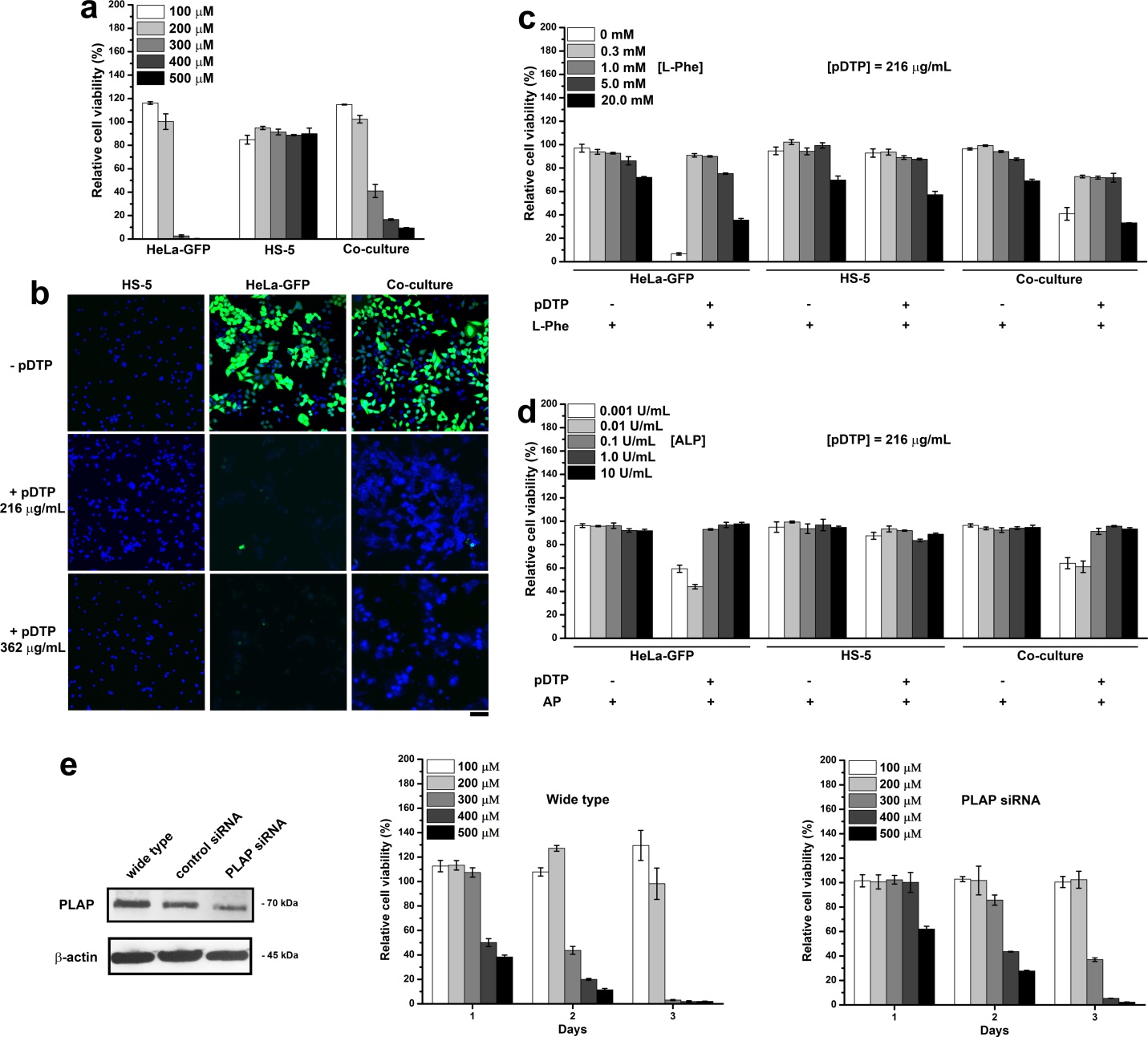


**Supplementary Figure 1. Selective inhibition of cancer cells.** a) Relative cell viability (determined by MTT assay; 100% represents the control, i.e., 0 μg/mL of the compound) of the HeLa-GFP, HS-5, or co-cultured cells (HS-5 and HeLa-GFP) incubated with pDTP at different concentrations. The incubation time is 48 hrs. The initial number of cells is 1.0×10^4^/well (i.e., 1.0×10^4^ HeLa-GFP or HS-5 cells, or mixture of 5.0×10^3^ HeLa-GFP cells and 5.0×10^3^ HS-5 cells). b) The confocal fluorescent images of separated or co-cultured cells incubated with or without pDTP (216 or 362 μg/mL) for 48 hrs. Blue indicates all the live cells stained by Hoechst 33342 (1 μg/mL, 5 min) prior to confocal fluorescent imaging; green indicates the live HeLa-GFP cells. The initial number of cells is 1.0×10^5^ per confocal dish. The scale bar is 100 μm. Relative cell viability of the HeLa-GFP, HS-5, or co-cultured cells incubated with c) L-Phe, pDTP + L-Phe; or d) AP, pDTP + AP in DMEM. [pDTP] = 216 μg/mL, [L-Phe] = 0, 0.3, 1.0, 5.0, 20.0 mM, [AP] = 0.001, 0.01, 0.1, 1.0, 10 U/mL. e) Western blot analysis shows change of PLAP in HeLa cells treated with wide type, control siRNA, and PLAP siRNA. Relative cell viability of wide type HeLa cells and PLAP siRNA HeLa cells treated with different concentrations of pDTP.


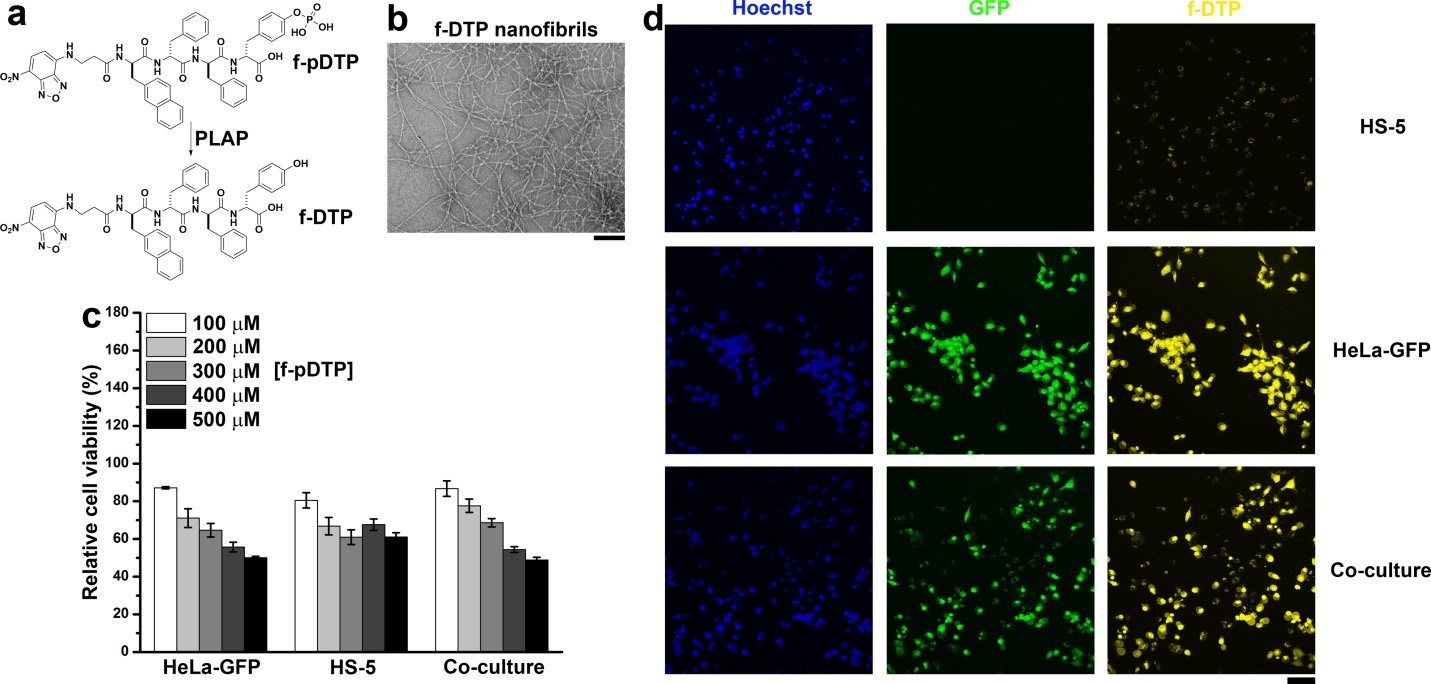


**Supplementary Figure 2. Imaging of DTP nanofibrils on cancer cells.** a) Chemical structures of the fluorescent precursor (f-pDTP), hydrogelator (f-DTP), and the dephosphorylation process catalyzed by PLAP. b) Transmission electron microscopic (TEM) images of f-DTP nanofibrils formed by the addition of AP (5 U/mL) to the solution of f-pDTP at the concentration of 493 μg/mL. The scale bar is 100 nm. c) The confocal fluorescent microscope images (×20 dry objective lens) show the fluorescence of the cells. The HeLa-GFP, HS-5, and co-culture of HeLa-GFP and HS-5 cells are incubated with 493 μg/mL f-pDTP in growth medium for 4 hrs. Blue indicates all the live cells stained by Hoechst 33342 at the concentration of 1 μg/mL; and green indicates the live GFP-HeLa cells; yellow indicates NBD staining. The initial number of cells is 1.0×10^5^ per confocal dish. The scale bar is 100 μm. d) Relative cell viability of the HeLa-GFP, HS-5, or co-cultured cells (HS-5 and HeLa-GFP) incubated with f-pDTP at different concentrations. The incubation time is 48 hrs. The initial number of cells is 1.0×10^4^/well.


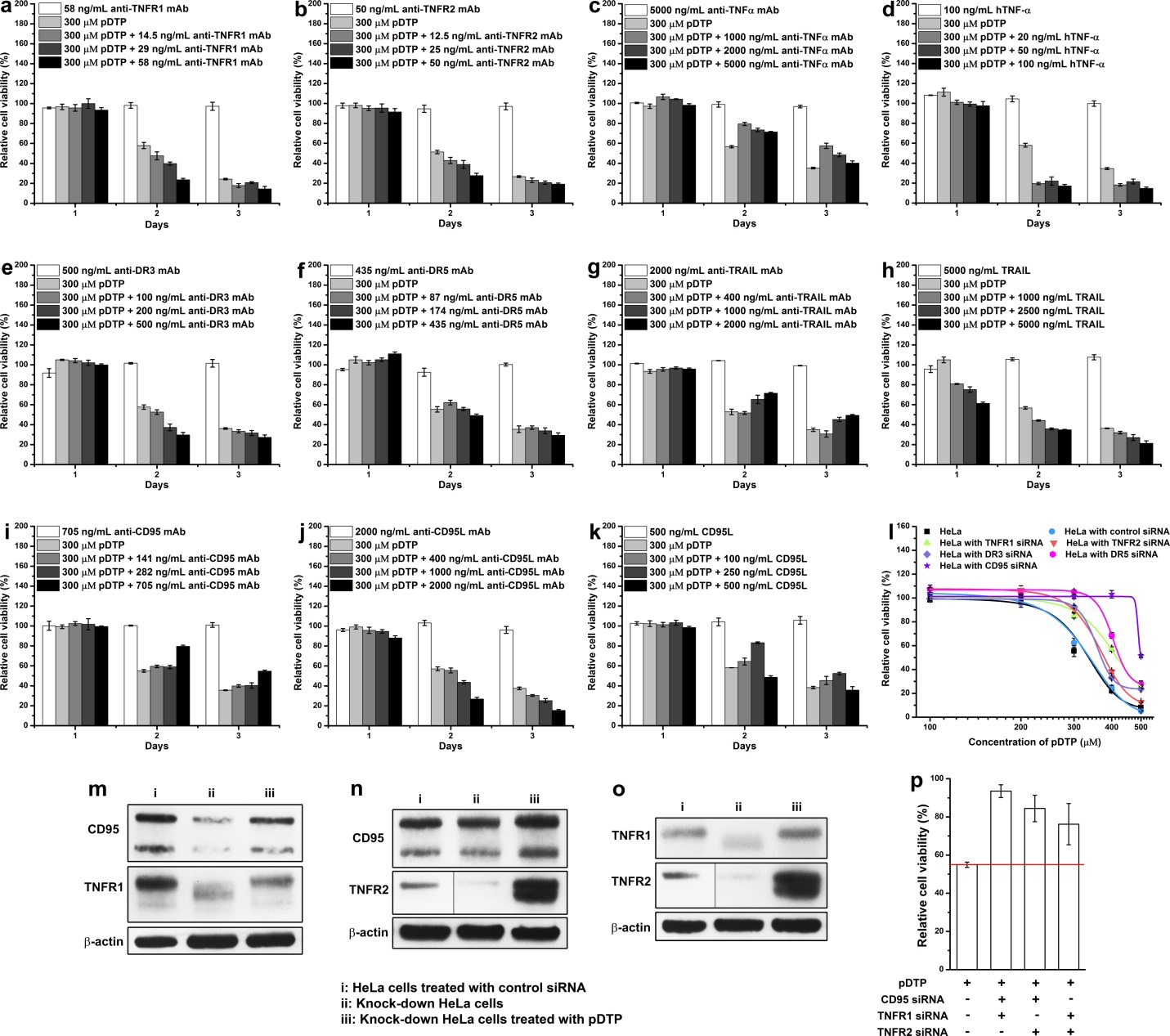


**Supplementary Figure 3. Viability of pDTP treated HeLa cells in the presence of cell death ligands or antibodies of cell death signaling molecules, or after knock-down of death receptors.** Relative cell viability of HeLa cells incubated with 300 μM pDTP and TNFα-related mAbs and ligand: a) anti-TNFR1, b) anti-TNFR2, c) anti-TNFα, or d) hTNF-α; TRAIL-related mAbs and ligand: e) anti-DR3, f) anti-DR5, g) anti-TRAIL, or h) TRAIL; CD95L-related mAbs and ligand: i) anti-CD95, j) anti-CD95L, or k) CD95L. l) Relative cell viability of HeLa or knock-down HeLa cells by siRNA incubated with different concentrations of pDTP for 48 hrs. The initial number of cells is 1.0×10^4^/well. Western blot shows the expression of death receptors (i.e., CD95, TNFR1, and TNFR2) in HeLa cells treated with m) CD95 and TNFR1 siRNA; n) CD95 and TNFR2 siRNA; o) TNFR1 and TNFR2 siRNA. p) Relative cell viability of HeLa cells or knock-down HeLa cells incubated with pDTP. The initial number of cells is 1.0×10^4^/well. n = 3.


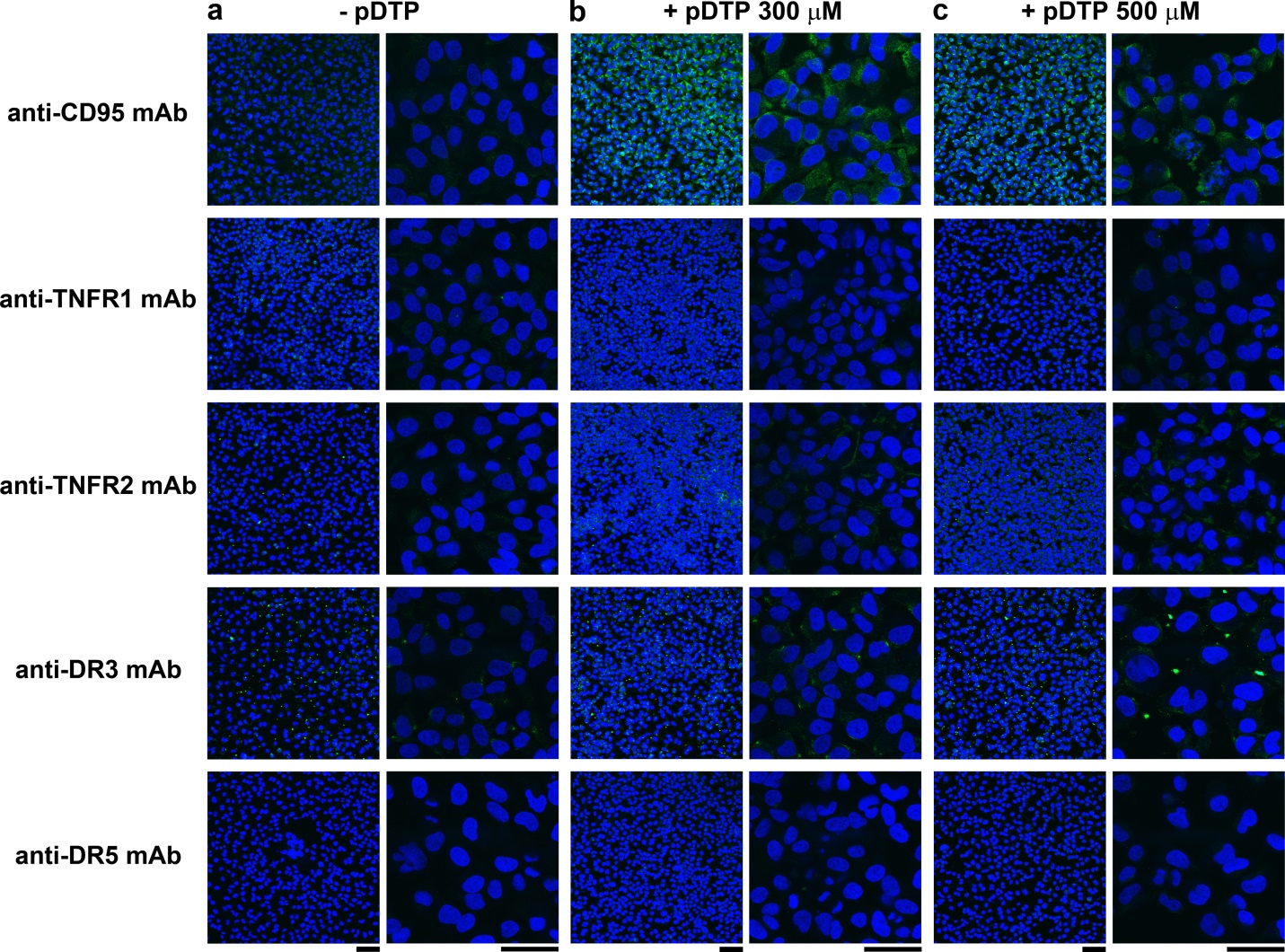


**Supplementary Figure 4.** **Imaging of cell death receptors in the presence of DTP nanofibrils**. The overlaid confocal fluorescent microscope images (×20 dry or ×100 oil objective lens) show the fluorescence of the cells. Cells are treated with cell death receptors antibodies (i.e., anti-CD95 mAb, anti-TNFR1 mAb, anti-TNFR2 mAb, anti-DR3 mAb, or anti-DR5 mAb) and a) growth medium (Minimum Essential Medium Eagle, control), b) 300 μM of pDTP, c) 500 μM of pDTP in growth medium. Blue indicates all the live cells stained by Hoechst 33342 at the concentration of 1 μg/mL; and green indicates the secondary antibodies. The initial number of cells is 1.0×10^6^/well. The scale bar is 100 μm for low magnification (Left) and 50 μm for high magnification (Right).


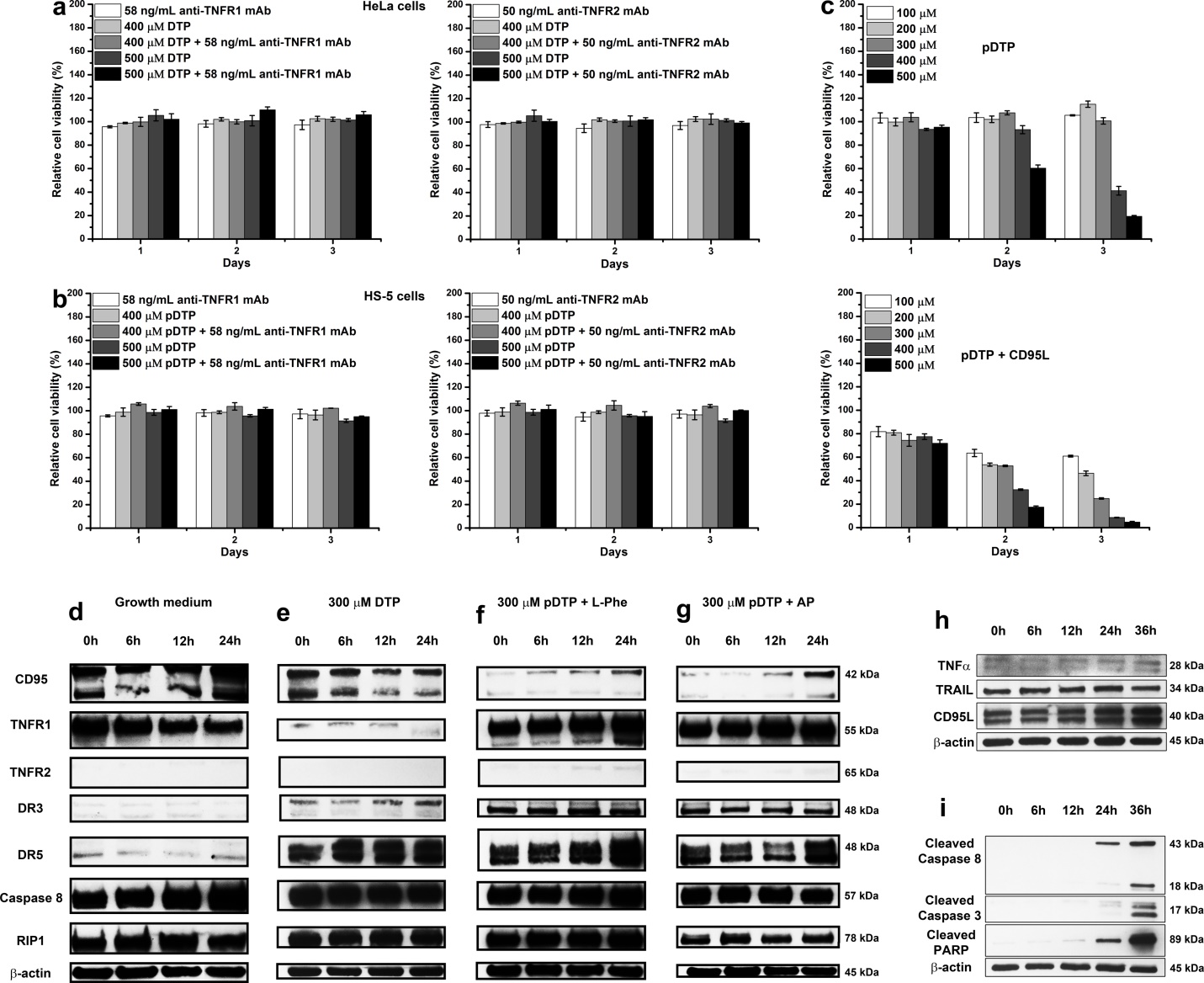


**Supplementary Figure 5.** **Controls and Western blot analysis**. Relative cell viability of a) HeLa cells incubated with 400 μM or 500 μM DTP and anti-TNFR1 mAb, or anti-TNFR2 mAb, or b) HS-5 cells incubated with 400 μM or 500 μM pDTP and anti-TNFR1 mAb, or anti-TNFR2 mAb. c) Relative cell viability of CD95L-KO HeyA8 cells incubated with a) pDTP, or b) the mixture of pDTP and 100 ng/mL CD95L. The initial number of cells is 1.0×10^4^/well. n = 3. Western blot analysis shows change of relative amount of several cell death receptor proteins and down-stream proteins over time in HeLa cells treated by d) growth medium; e) 300 μM of DTP; f) 300 μM of pDTP and 5 mM L-Phe; g) 300 μM of pDTP and 5 U/mL AP. Western blot shows change of relative amount of h) the cell death ligands; or i) several cell death related proteins, over time in HeLa cells treated by pDTP at the concentration of 300 μM.


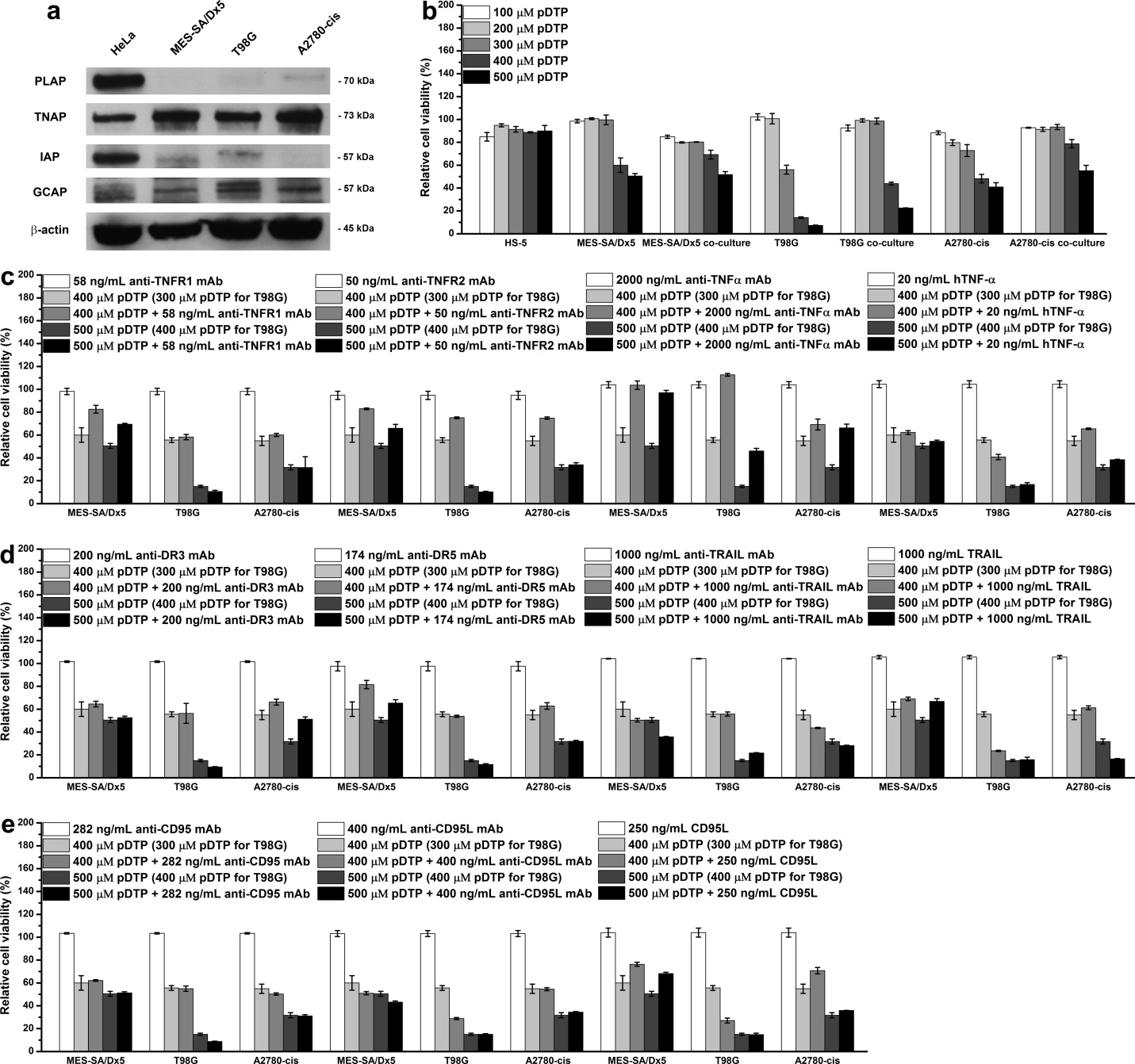


**Supplementary Figure 6.** **Viability of pDTP treated MDR cells in the presence of cell death ligands or antibodies of cell death signaling molecules.** a) Western blot analysis shows expression of four kinds of phosphatase isoforms on membrane of four kinds of cell lines. b) Relative cell viability of HS-5 cells, MES-SA/Dx5, T98G, A2780-cis cells, and co-culture of cancer and HS-5 cells incubated with pDTP at different concentrations. The initial number of co-culture is 5.0×10^3^ cancer cells and 5.0×10^3^ HS-5 cells for each well. Relative cell viability of MES-SA/Dx5 cells incubated with 400 μM or 500 μM pDTP; T98G cells incubated with 300 μM or 400 μM pDTP; A2780-cis cells incubated with 400 μM or 500 μM pDTP and d) TNFα-related mAbs; e) TRAIL-related mAbs; or f) CD95L-related mAbs. The incubation time is 48 hrs. The initial number of homogeneous cells is 1.0×10^4^/well.


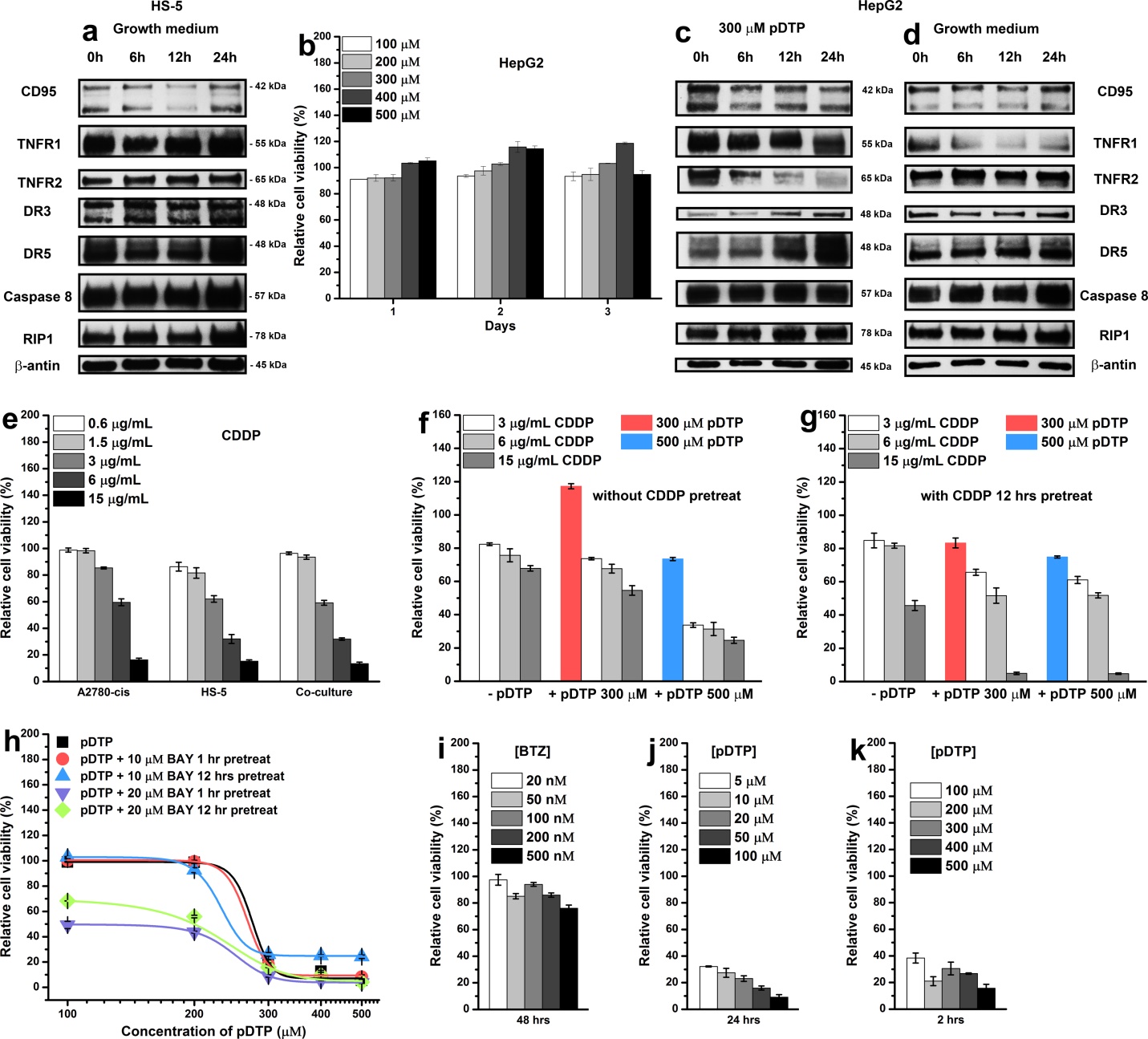


**Supplementary Figure 7.** **Controls and Combinations**. Western blot analysis shows change of relative amount of several cell death receptor proteins and down-stream proteins over time in a) HS-5 cells treated by growth medium or c, d) in HepG2 cells treated with c) 300 μM of DTP, d) growth medium.; Relative cell viability of b) HepG2 cells treated with pDTP at different concentrations; e) A2780-cis, HS-5, and co-culture of A2780-cis and HS-5 cells incubated with CDDP at different concentrations for 24 hrs; A2780-cis cells incubated with pDTP and CDDP of different concentrations for 24 hrs f) without CDDP pretreatment or g) with CDDP pretreatment for 12 hrs; h) HeLa cells incubated with pDTP and BAY that pretreats cells for 1 or 12 hrs; HeLa cells incubated with i) BTZ for 48 hrs, j) pDTP of lower concentrations and 20 µM BAY for 24 hrs, or k) pDTP of different concentrations and 20 µM BAY for 2 hrs. The initial number of cells is 1.0×10^4^/well.


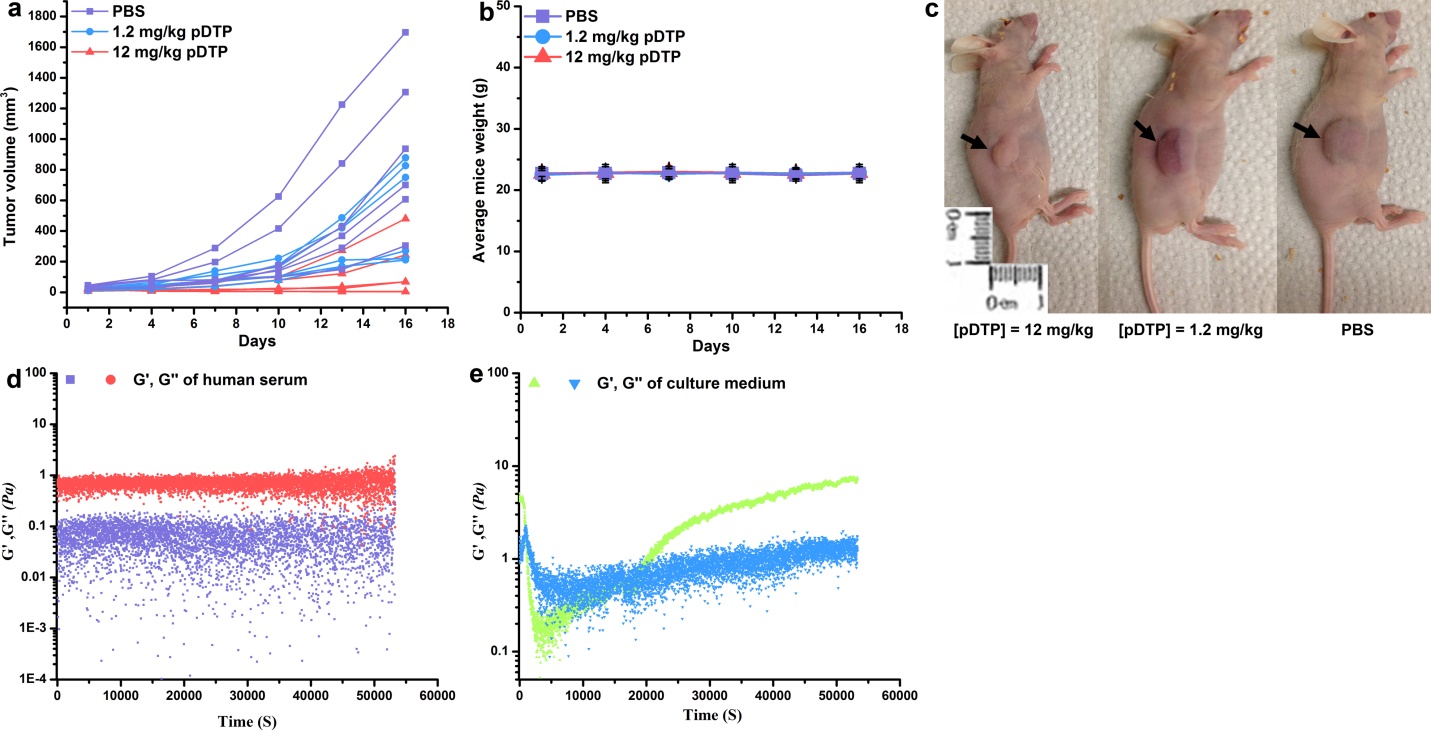


**Supplementary Figure 8. DTP fibrils inhibit MES-SA/Dx5 tumor on nu/nu mice.** a) Tumor progression curves of each mouse bearing MES-SA/Dx5 tumors. 0.1 mL of pDTP at 1.2, 12 mg/kg or just PBS buffer as control is injected subcutaneously and peritumorally in every three days (five doses, starting day 1). b) The change of the body weights of mice during the treatment. c) Representative image shows mice bearing tumors with similar initial volume from each group on 16^th^ day of treatment. Black arrows point at tumor. Time dependent rheometry shows the dynamic storage moduli (G’) and the loss moduli (G”) change of 362 μg/mL pDTP in d) the human serum; e) the conditioned medium of HeLa cells (We obtain the conditioned medium by incubating 1.0×10^6^ HeLa cells in the normal growth medium (MEM with FBS and antibiotics) for 24 hrs).


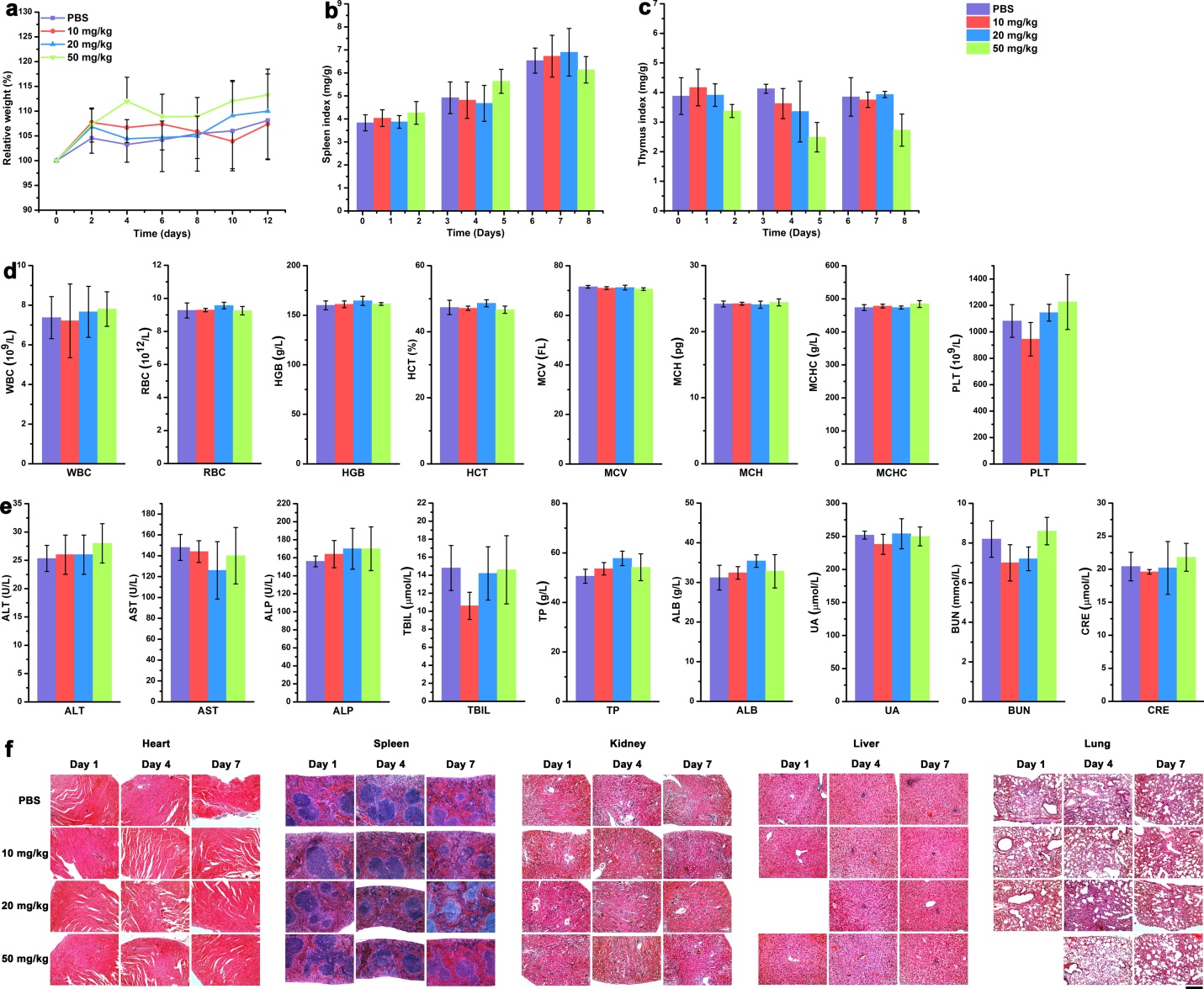


**Supplementary Figure 9. *In vivo* compatibility of pDTP in mice.** a) The change of the body weights of nu/nu mice during the treatment. 0.1 mL of pDTP at 10, 20, 50 mg/mL, or just PBS buffer as control is injected intravenously (starting day 0), and then the body weights of mice are tested every two days. The change of the b) spleen or c) thymus index over time. d) Blood test parameters or e) blood biochemistry test of the nu/nu mice are measured. f) Haematoxylin and eosin (H&E) staining images of heart, spleen, kidney, liver, and lung from the nu/nu mice. The scale bar is 100 µm.

**Supplementary Table 1.** IC_50_ values for pDTP in human cell lines.

| Cell lines | IC_50_ µM (µg/mL) | |
| --- | --- | --- |
|  | 48 hrs | 72 hrs |
| HeLa  (cervical adenocarcinoma) | 289 (209) | 258 (187) |
| HeLa-GFP  (cervical adenocarcinoma) | 238 (172) | 240 (174) |
| HS-5  (normal) | >500 (362) | >500 (362) |
| T98G  (glioblastoma multiforme) | 310 (224) | 281 (203) |
| MES-SA/Dx5  (uterine sarcoma) | 500 (362) | 500 (362) |
| A2780-cis  (ovarian carcinoma) | 405 (293) | 292 (211) |
| SK-OV-3  (ovarian adenocarcinoma) | >500 (362) | >500 (362) |
| Saos-2  (osteosarcoma) | 295 (213) | 252 (182) |
| HepG2  (hepatocellular carcinoma) | >500 (362) | >500 (362) |
| MCF-7  (breast adenocarcinoma) | 403 (291) | 390 (282) |
| HCC1937  (primary ductal carcinoma) | >500 (362) | >500 (362) |
| A-375  (malignant melanoma) | >500 (362) | 394 (285) |
| U-87 MG  (glioblastoma; astrocytoma) | 423 (306) | 304 (220) |

Cells are incubated for 48 or 72 hrs in the presence of increasing concentrations of pDTP (range 100-500 µM, or 72-362 µg/mL). Cell viability is evaluated with the MTT assay (see Methods). The concentrations able to inhibit 50% viability (IC_50_) are calculated and the initial number of cells is 1.0×10^4^/well.
